# Sustainable Recovery of Cu, Fe, Ni, and Zn from Multilayer Ceramic Capacitors Using a Ternary Deep Eutectic Solvent

**DOI:** 10.3390/molecules30214254

**Published:** 2025-10-31

**Authors:** Jordy Masache-Romero, Katherine Moreno, Fernando Sánchez, Carlos F. Aragón-Tobar

**Affiliations:** 1 Department of Extractive Metallurgy, Escuela Politécnica Nacional, Ladrón de Guevara E11-253, P.O. Box 17-01-2759, Quito 170525, Ecuador; jordy.masache@epn.edu.ec (J.M.-R.); katherine.moreno@epn.edu.ec (K.M.); 2 Department of Materials, Escuela Politécnica Nacional, Ladrón de Guevara E11-253, P.O. Box 17-01-2759, Quito 170525, Ecuador; fernando.sanchez01@epn.edu.ec

**Keywords:** choline chloride–citric acid–glycerol deep eutectic solvent, multilayer ceramic capacitors, electronic waste (e-waste), critical metals

## Abstract

The rapid growth in electronic waste (e-waste) generation highlights the urgent need for efficient and environmentally sustainable methods for metal recovery. This study focuses on the selective recovery of valuable metals from multilayer ceramic capacitors (MLCCs), commonly found in printed circuit boards (PCBs) of post-consumer electronics. MLCCs were manually recovered from dismantled computer PCBs, thermally treated, pulverized, and characterized using X-ray fluorescence and X-ray diffraction techniques. To evaluate green alternatives to traditional acid leaching, three deep eutectic solvents (DESs) based on choline chloride (ChCl) were prepared: citric acid (CA), glycerol (GLY), and a ternary (GLY-CA) mixture of both (GLY-CA). Leaching experiments were conducted over a 24 h period and analyzed using atomic absorption spectroscopy. The results showed complete recovery (100%) of copper using both CA and the GLY-CA mixture, while nickel recovery reached 100% with CA and moderate levels with GLY-CA. Zinc recovery was also high (99%) with both CA and GLY-CA. Iron showed a maximum recovery of 60%, potentially due to its occurrence in various chemical forms. The ternary DES (GLY-CA) demonstrated lower viscosity, improving handling and operational efficiency. These findings highlight the potential of citric-acid-based and ternary (GLY-CA) DESs as effective, low-toxicity leaching agents for the recovery of critical metals from MLCCs.

## 1. Introduction

The accelerated pace of technological innovation and the growing global consumption of electronic devices have led to a dramatic increase in the generation of waste electrical and electronic equipment (WEEE), reaching an estimated 62 million tons in 2022, and many of these devices reach the end of their useful life within increasingly shorter periods due to rapid technological obsolescence and changing consumer habits [1]. Although WEEE contains valuable metals such as copper, silver, nickel, and zinc, a large proportion is not treated correctly, resulting in both significant environmental impacts and the loss of critical resources [2]. This situation presents an urgent challenge for waste management systems, which must develop more efficient and sustainable processes for recovering these materials.

Among the components commonly found in printed circuit boards (PCBs), multilayer ceramic capacitors (MLCCs) are particularly noteworthy due to their ubiquity and composition [3]. These devices, made up of alternating layers of ceramic dielectric and metallic electrodes, serve essential functions in electronic circuits by efficiently storing and releasing electrical energy in a compact volume, and from a materials recovery perspective, MLCCs are a significant source of strategic metals, such as nickel, copper, silver, and zinc, as well as advanced ceramic materials like barium titanate (BaTiO_3_), often doped with oxides of manganese, yttrium, holmium, and others [4]. Despite their high metal content, MLCCs are typically overlooked or processed together with other waste streams, which limits the potential for selective and efficient recovery.

Currently, two main metallurgical approaches dominate the recovery of metals from solid waste: pyrometallurgy and hydrometallurgy; pyrometallurgical methods involve high-temperature processes such as roasting and smelting, which can achieve high metal purity but often at the cost of significant energy consumption and toxic emissions, particularly when plastics or other organic compounds are present [1]. In contrast, hydrometallurgical techniques, especially acid leaching, offer a lower environmental impact and greater selectivity by operating at moderate temperatures. However, conventional leaching agents such as sulfuric, nitric, and hydrochloric acids are highly corrosive, hazardous, and difficult to manage safely [5]. Given these limitations, more sustainable alternatives have been proposed in recent years, including the use of carboxylic acids [1]. Acids such as acetic, oxalic, and citric have demonstrated recovery efficiencies of up to 90% for metals like copper, nickel, and zinc from e-waste [6].

Among the emerging leaching agents, deep eutectic solvents (DESs) have gained significant attention due to their low toxicity, biodegradability, and tunable properties, making them a promising alternative for metal recovery from e-waste [7]. DESs are formed from mixtures of hydrogen bond donors (HBDs) and hydrogen bond acceptors (HBAs) that produce a eutectic mixture with a melting point lower than that of their individual components [8]. Unlike traditional solvents, DES can be tailored to specific applications by adjusting its composition, offering enhanced selectivity and efficiency in the leaching process [7]. They are generally biodegradable, non-volatile, and less toxic. They can be synthesized from inexpensive, readily available materials such as choline chloride, urea, citric acid, glycerol, ethylene glycol, and even sugar like glucose [9].

Applications of DESs for metal recovery from a wide range of materials have gained significant attention in recent years. For example, Aragón-Tobar et al. [10] demonstrated the effectiveness of three widely used choline chloride-based DESs, Reline (ChCl:urea, 1:2), Ethaline (ChCl:ethylene glycol, 1:2), and Glyceline (GLY) (ChCl:glycerol, 1:2), in leaching Cu, Fe, Pb, and Zn from synthetic oxides, synthetic sulfates, and natural sulfides. Moreno et al. [11] applied similar DES formulations to a sphalerite–galena concentrate, a primary source; their findings underscore the ability of DESs to handle mineral matrices with complex compositions, especially when combined with oxidants like iodine and thermal pretreatments. Salgado et al. [12] further extended the use of these same DESs to recover Pb from metallurgical slag, illustrating their potential in treating complex secondary residues. These studies collectively highlight the versatility of DESs in processing both primary and secondary sources. However, these are not the only studies available; they are representative of the types of sources investigated to date, including oxides, sulfates, sulfides, mineral concentrates, and metallurgical slags, demonstrating the adaptability of DESs across diverse matrices. The application of DESs in the recycling of electronic waste, a highly heterogeneous and increasingly important secondary resource, is an emerging field that, despite recent progress, still requires substantial research and innovation. Given the complex composition of e-waste and the limitations of conventional leaching agents, the use of DESs for metal recovery from this type of waste presents an appealing and promising alternative [7].

The extraction of various metals (Cu, Fe, Ni, Zn, Ag, Au, Al) from printed circuit boards (PCBs) has been successfully achieved using deep eutectic solvents (DESs) formulated with choline chloride as the hydrogen bond acceptor (HBA), combined with different hydrogen bond donors (HBDs). The HBDs employed include ethylene glycol [7,13,14,15,16,17,18], glycerol [19,20], citric acid [13,19], malonic acid [13,15], oxalic acid [17,21,22], acetic acid [13], chloroacetic acid [23,24], glycolic acid [17], formic acid [22], levulinic acid [13], and lactic acid [21].

A ternary solvent is a homogeneous mixture composed of three components, in which their interactions yield a liquid with physical and chemical properties distinct from those of each component or their binary combinations. In the context of deep eutectic solvents (DESs), ternary solvents typically comprise a base, such as choline chloride, and two hydrogen bond donors (HBDs) [25,26]. This combination enables precise tuning of properties such as viscosity, melting point, thermal stability, and solvation capacity, thereby facilitating their application in sustainable and targeted processes [27].

Ternary deep eutectic solvents (TDESs) offer several advantages over their binary counterparts (BDESs) in terms of operating conditions and selectivity, mainly due to the ability to fine-tune and optimize their properties through the incorporation of a third component [28].

This modification enables a reduction in viscosity, an increase in thermal stability, and an improvement in solubility, thereby facilitating more efficient processes with lower energy consumption [27]. Furthermore, TDESs demonstrate a greater capacity for selectively targeting specific metals, as the third component can be tailored to enhance affinity toward particular metal ions, thus increasing both efficiency and precision in metal separation and recovery processes [19]. These improvements in operating conditions and selectivity make TDESs more versatile and adaptable for sustainable industrial applications compared to traditional binary solutions.

Despite this promising progress, the application of DESs to recover metals from multilayer ceramic capacitors (MLCCs) remains unexplored. While some studies have examined the use of DESs in treating electronic waste, their direct application to selectively extracted MLCCs remains scarce. This gap not only limits the development of targeted recycling strategies but also contributes to the ongoing underutilization of valuable materials embedded in electronic components. Furthermore, most of the reported work has focused on binary DESs (BDESs), which, although effective, often present limitations such as high viscosity and reduced selectivity [19].

These features make TDESs a more versatile alternative, with the potential to overcome the operational and selectivity limitations of binary systems, thereby opening new avenues for efficient and sustainable MLCC recycling.

Therefore, this study aims to evaluate the performance of a ternary deep eutectic solvent, composed of choline chloride, glycerol, and citric acid, as a sustainable leaching agent for the selective recovery of valuable metals (Cu, Fe, Ni, Zn) from multilayer ceramic capacitors. By addressing the technical efficiency of this alternative solvent, this work aims to advance the development of safer, more efficient, and eco-friendly recycling processes for critical electronic components, thereby contributing to the broader goals of resource recovery and a circular economy within the context of electronic waste management.

## 2. Results

### 2.1. Chemical and Crystalline Characterization of the Multilayer Ceramic Capacitors

#### 2.1.1. Chemical Characterization

Table 1 shows the results of the chemical characterization of the material used in this research, obtained through X-ray fluorescence (XRF).

The elemental composition of the MLCC is presented in Table 1. The principal constituent is barium (Ba), accounting for 45.8% of the total content. Other significant elements include nickel (Ni) at 16.1%, titanium (Ti) at 14.8%, and iron (Fe) at 5.8%. Minor components include tin (Sn) (3.8%), copper (Cu) (2.5%), and zinc (Zn) (1.5%). The remaining 9.7% corresponds to other minor elements.

Based on the elemental composition previously described, barium (~46%) and titanium (~15%) are the predominant elements identified in the sample, forming the primary ceramic matrix characteristic of the MLCC. In this case, the ceramic phase is based on Ba and Ti, and it functions as the primary energy-storage medium through polarization under an applied electric field. Moreover, it acts as an electrical insulator, preventing the flow of direct current between the electrodes while allowing only the accumulation and rapid release of charge [29].

Certain elements are incorporated into the ceramic to enhance properties. Among them, zinc (Zn) acts as a dopant within the BaTiO_3_-based dielectric ceramic, introduced in the form of zinc oxide (ZnO) to refine the microstructure, control sintering, and enhance dielectric stability. In addition to its role in ceramics, zinc is also commonly found in metal alloys, most notably with copper, where its incorporation improves corrosion resistance, thereby contributing to the long-term preservation of electrical conductivity in conductive components [29,30].

Iron (Fe) is not a significant component of the ceramic matrix but may occur as a dopant or as a process-related impurity, potentially influencing the dielectric’s electrical performance. Iron is not a primary conductive metal like copper; instead, it is associated with alloys and coatings used in connectors and solder joints and is also found in specific electronic components, such as the cores of inductors and transformers. Its presence in PCBs is more closely related to metallic support structures and to alloys combined with copper, nickel, or tin. Iron is mainly located in metallic reinforcement and shielding layers, solder alloys and external terminations (together with Ni, Sn, and Cu), as well as in some magnetic components soldered onto PCBs (inductors, cores) [30].

In addition to these ceramic components, nickel (~16%), tin (~3.8%), and copper (~2%) are commonly found in the metallic layers of MLCCs. Nickel and copper are typically used in the internal electrodes, often in combination with silver or palladium alloys, depending on the specific capacitor design [3]. In fact, according to Lu et al. [29], copper (Cu) functions as a conductor, replacing palladium and silver in modern MLCC designs, and is primarily found in the metallic electrode sections. Moreover, the external terminations of MLCCs are generally coated with layers of nickel (Ni) and tin (Sn) to enhance electrical conductivity and solderability during mounting and integration into electronic circuits [30]. These findings are consistent with those reported by Laadjal & Cardoso [31], who demonstrated that both Ni and Sn are incorporated into MLCCs to enhance electrical conductivity while lowering production costs, as they can substitute for the more expensive noble metals palladium (Pd) and platinum (Pt).

#### 2.1.2. Crystalline Phase Characterization

The composition listed in Table 2 corresponds to the crystalline fraction of the MLCCs, identified through X-ray diffraction (XRD). Amorphous phases or minor components below the detection limit are not represented.

Table 2 presents the results of the crystalline phase characterization of the analyzed material. This table identifies the main compounds present in the sample, highlighting the phases that constitute the ceramic matrix as well as the metallic components associated with the internal electrodes and external terminations. The analysis of MLCCs by X-ray diffraction (XRD) reported results expressed in abundances rather than weight percentages. This condition arises because the sample does not exhibit complete crystallization. Such an amorphous component does not produce well-defined peaks in the diffractogram but instead generates a diffuse background signal, which prevents an absolute quantification of the phases present.

The principal constituent of the crystalline fraction of MLCCs is barium titanate (BaTiO_3_), identified as the predominant phase within the ceramic matrix. This compound serves as the primary dielectric material in MLCCs due to its ferroelectric behavior and high dielectric constant (high-k), which enable the achievement of high capacitance values within reduced volumes [31].

In addition, the presence of barium iron oxide (BaFeO_3−x_) was identified as the second ceramic constituent of the MLCCs, associated with barium titanate. This phase is of particular importance, as iron incorporation can partially substitute either titanium or barium within the barium titanate perovskite, a material widely recognized for its high dielectric permittivity and low dielectric loss [32].

The presence of magnetite (Fe_3_O_4_) was also identified, which, according to Sideris et al. [3], is not an intentional design constituent of MLCCs. Its occurrence reflects the incorporation of excess iron and, in some cases, is associated with the formation of iron-based compounds arising from dopants or impurities.

The copper–zinc phase (CuZn_5_) was also identified, although in lower abundance. According to Laadjal & Cardoso [31], this alloy acts as a conductive phase primarily employed in the internal electrodes of MLCCs. The incorporation of zinc into copper enhances corrosion resistance, thereby contributing to the long-term preservation of electrical conductivity. The CuZn_5_ alloy is predominantly located within the metallic conductive section of MLCCs [33]. Additionally, Laadjal & Cardoso [31] also indicate an additional origin of zinc, suggesting that trace amounts may also reside within the ceramic matrix of MLCCs.

### 2.2. Leaching Test with Deep Eutectic Solvents

#### 2.2.1. Copper Leaching

**Figure 1:**
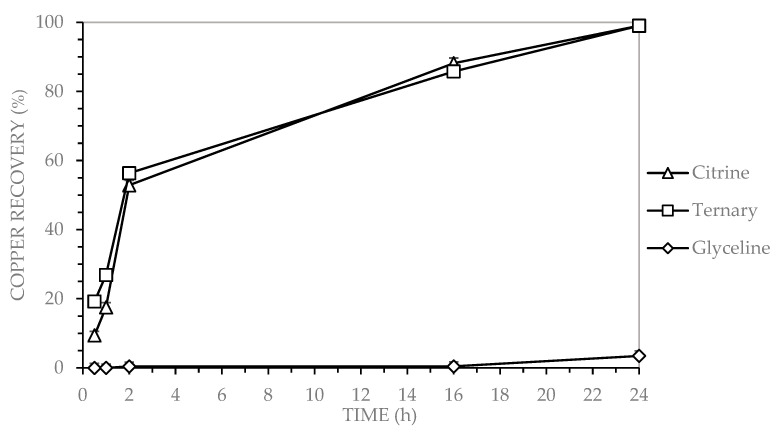
Copper recovery by leaching MLCCs using three DESs (glyceline (GLY), citrine (CA), and ternary), under the following operating conditions: 2% solids content, 400 rpm stirring speed, 90 °C, for a duration of 24 h.

Figure 1 shows that copper recovery from MLCCs was evaluated using three deep eutectic solvents (DES): glycine (choline chloride and glycerol), citrine (CA) (choline chloride and citric acid), and a ternary system (GLY-CA) (choline chloride, glycerol, and citric acid).

As presented in Figure 1, the ternary DES (GLY-CA), composed of choline chloride, glycerol, and citric acid, achieved a cooper recovery of 99% after 24 h. A similar result (99% recovery) was obtained with citrine (CA) (choline chloride and citric acid) under the same operating conditions. In contrast, glyceline (GLY) (choline chloride and glycerol) displayed a lower recovery with only 3% after 24 h. Both citrine (CA) and the ternary DES (GLY-CA) exceeded 50% recovery within the first 2 h of leaching, progressively reaching approximately 99% after 24 h. Therefore, copper from MLCC was effectively recoverable with ternary (GLY-CA) and citrine (CA) DES systems under the tested conditions.

#### 2.2.2. Nickel Leaching

**Figure 2:**
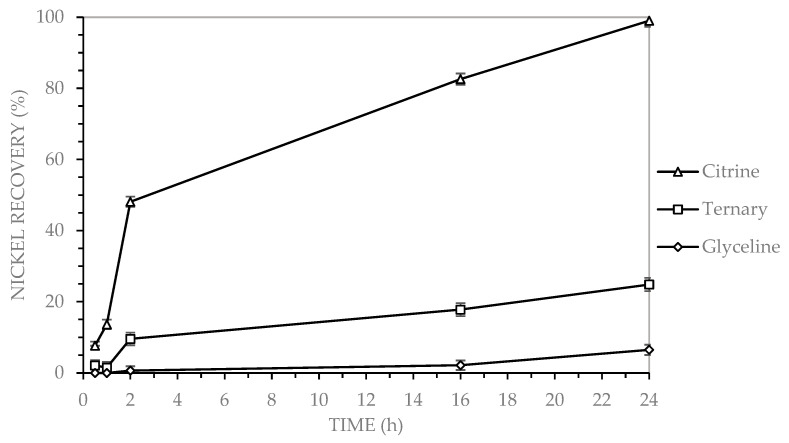
Nickel recovery by leaching MLCCs using three DESs, under the following operating conditions: 2% solids content, 400 rpm stirring speed, 90 °C, for a duration of 24 h.

Figure 2 shows that nickel recovery from MLCCs was evaluated using three deep eutectic solvents (DES): glycine (choline chloride and glycerol), citrine (CA) (choline chloride and citric acid), and a ternary system (GLY-CA) (choline chloride, glycerol, and citric acid).

Figure 2 illustrates that after 24 h, citrine (CA) facilitated a nickel recovery of 99%, in contrast to the ternary (GLY-CA) and glyceline (GLY), which yielded recoveries of approximately 25% and 6%, respectively. Citrine (CA) achieved a nickel recovery of 38% after just 2 h and progressively reached 99% by 24 h. The ternary DES (GLY-CA) exhibited an initial recovery of 39% within the first hour, followed by a slight decrease to 37% after 16 h, and ultimately attained a recovery of 68% at 24 h. In contrast, glyceline (GLY) demonstrated lower performance, with only 2% recovery after 2 h, increasing to 5% at 16 h, and reaching a final value of 16% after 24 h.

#### 2.2.3. Iron Leaching

**Figure 3:**
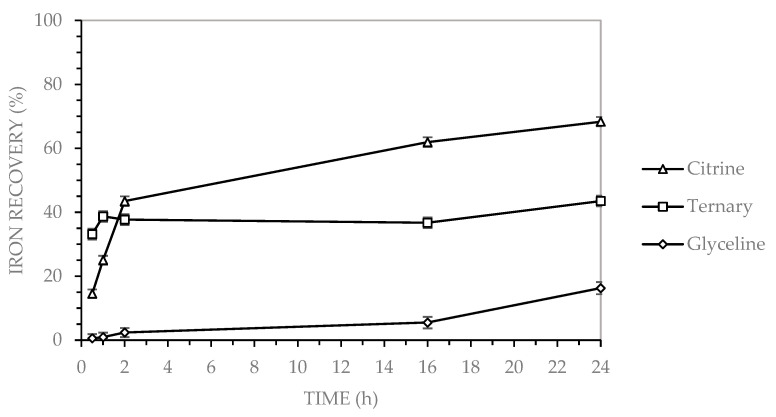
Iron recovery by leaching MLCCs using three DESs, under the following operating conditions: 2% solids content, 400 rpm stirring speed, 90 °C, for a duration of 24 h.

Figure 3 shows that iron recovery from MLCCs was evaluated using three deep eutectic solvents (DES): glycine (choline chloride and glycerol), citrine (CA) (choline chloride and citric acid), and ternary DES (GLY-CA) (choline chloride, glycerol, and citric acid).

Figure 3 shows that iron recovery exhibited a distinct behavior among the three DESs, differing from the trends observed in copper and nickel. Citrine (CA) reached approximately 45% recovery within the first 2 h and continued to increase, attaining nearly 70% after 24 h. The ternary DES (GLY-CA) achieved an initial recovery of approximately 40% after 2 h, but subsequently remained relatively constant, reaching only about 42% by the end of the test (24 h). In contrast, glyceline (GLY) showed the lowest performance, with only 18% after 24 h.

#### 2.2.4. Zinc Leaching

**Figure 4:**
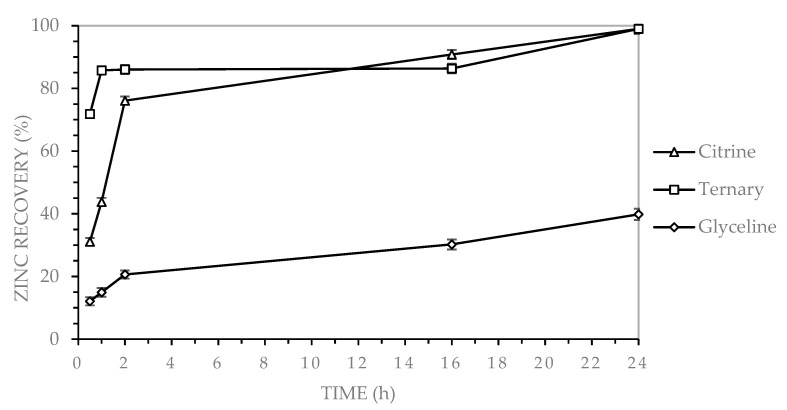
Zinc recovery by leaching MLCCs using three DESs, under the following operating conditions: 2% solids content, 400 rpm stirring speed, 90 °C, for a duration of 24 h.

Figure 4 shows that zinc recovery from MLCCs was evaluated using three deep eutectic solvents (DES): glycine (choline chloride and glycerol), citrine (CA) (choline chloride and citric acid), and a ternary system (GLY-CA) (choline chloride, glycerol, and citric acid).

As shown in Figure 4, the ternary DES (GLY-CA) rapidly reached values close to 90% within the first hour, remaining nearly constant and achieving approximately 95–100% after 24 h. Similarly, citrine (CA) reached around 70% at the first hour and increased up to nearly 100% by the end of the test. In contrast, glyceline (GLY) showed minimal performance, with only 20% recovery in the early stages and a maximum of about 40% after 24 h, confirming that the presence of citric acid is essential for efficient zinc dissolution.

## 3. Discussion

### 3.1. Multilayer Ceramic Capacitors as a Source of Copper, Iron, Nickel, and Zinc

While traditionally extracted from mined ores, many valuable metals are now found in higher concentrations within electronic waste, particularly in multilayer ceramic capacitors. These components, often overlooked, can rival or even surpass the metal content of primary mineral resources.

The results obtained in this study show that MLCCs exhibit a significantly enriched metallic composition compared to traditional primary sources. Characterization revealed contents of 16% nickel, 5.8% iron, 2.5% copper, and 1.5% zinc, values that are notably higher, in most cases, than those reported for ores and natural deposits.

In the case of copper, world-class deposits, such as Escondida in Chile, which is regarded as the largest producer globally, display grades that do not exceed 1% Cu in sulfide matrices [34]. An MLCC with a copper content of 2.5% contains twice as much copper as this natural sulfide deposit, despite having a less complex matrix and with copper present in metallic form rather than as sulfides. Similarly, for nickel, the Voisey’s Bay deposit in Canada exhibits average grades of 1.59% Ni, primarily hosted in sulfides such as pentlandite [35]. In contrast, MLCCs reach up to 16% Ni, positioning them as a secondary resource of exceptionally high metal concentration.

Regarding zinc, the Red Dog deposit in Alaska (USA), one of the leading global producers, represents a polymetallic Zn-Pb-Ag deposit hosted in black shales of the Kuna Formation. In this district, average reserve grades with zinc content close to 16% have been reported [36]. This value is higher than the 1.5% Zn observed in MLCCs; however, it should be noted that in these electronic devices, a portion of this metal is contained in a concentrated ceramic matrix, which is relatively accessible for recovery processes.

In the case of iron, the comparison is markedly different. The Carajás deposit in Brazil, formed in an Archean context (~2.7 Ga), constitutes an exceptional banded iron formation (BIF) deposit with grades exceeding 65% Fe [37]. These values, which represent unique geological concentrations at a global scale, far surpass the 5.8% Fe identified in MLCCs. Nevertheless, the relevance of iron in electronic waste does not lie in achieving grades comparable to those of giant deposits such as Carajás, but rather in the possibility of simultaneous recovery alongside other strategic metals.

Overall, this contrast highlights that, aside from exceptional cases such as the Carajás iron deposits, MLCCs constitute an extraordinarily rich secondary source of strategic metals, particularly nickel and copper, whose grades far exceed those of conventional primary ores. Although these components are typically processed together with printed circuit boards, the selective separation of MLCCs could represent a promising approach to enable more focused and efficient metal recovery.

### 3.2. Copper, Nickel, Iron, and Zinc Recovery in Binary Deep Eutectic Solvents: A Comparison with Previous Studies

#### 3.2.1. Copper Recovery in Binary DES

A review of the literature revealed no prior studies employing the same deep eutectic solvents (DESs) evaluated in the present work, nor were any investigations identified that utilize a ternary DES for this application. Similarly, no studies were found that focus specifically on multilayer ceramic capacitors (MLCCs); therefore, comparative references are drawn from research on printed circuit boards (PCBs), which commonly contain MLCCs.

For ChCl-based carboxylic-acid DESs, both the identity of the hydrogen-bond donor (HBD) and the use of an oxidant strongly affect Cu dissolution kinetics and overall yields. Oke et al. [23] employed choline chloride with trichloroacetic acid and H_2_O_2_, achieving ~100% Cu recovery at 65 °C in 3 h, lower temperature and shorter time than the baseline presented in this study, indicating that oxidant-assisted pathways could accelerate Cu recovery. Saffaj et al. [13] reported a yield of ~99% Cu using ChCl–acetic acid and I_2_ at 65 °C for 96 h, i.e., a longer residence time at a moderate temperature. Mishra et al. [22] obtained ~90% Cu with ChCl–formic acid at 100 °C for 16 h, which is comparable to the results of this study, where ~86% Cu was recovered at 90 °C for 16 h. In contrast, Domańska et al. [15] reported ~16% Cu for ChCl–malonic acid at 60 °C for 2 h with H_2_O_2_, whereas in this study, citric-acid (CA)–based DES reached ~50% under analogous conditions, suggesting a more favorable performance of CA relative to malonic acid. Liu et al. [16] achieved ~22% Cu at 25 °C in 25 min using ChCl–oxalic acid with H_2_O_2_, illustrating rapid but limited extraction at ambient conditions. From an oxidant-effect perspective, Emmons-Burzyńska et al. [21] further corroborate the positive role of H_2_O_2_ by reporting enhanced Cu recoveries in lactic-acid systems.

Collectively, these comparisons (i) underscore the novelty of the present work regarding both the use of ternary DESs and the focus on MLCCs; (ii) highlight the sensitivity of performance to HBD identity within ChCl-based DESs; and (iii) demonstrate the beneficial and consistent impact of oxidants (e.g., H_2_O_2_, I_2_) on both the rate and extent of metal dissolution, an operational lever that can be exploited to further optimize the ternary DES investigated here.

In the case of glycerin-based deep eutectic solvents (DESs), lower metal recovery rates were observed. According to the reviewed literature, this type of DES has primarily been associated with silver recovery from printed circuit boards (PCBs) [15,19,20]. Lu et al. [29] reported a copper recovery of 11% using a glycerin-based DES under conditions of 25 °C and 25 min, in the presence of hydrogen peroxide as an oxidizing agent. This result highlights the beneficial role of the oxidant in enhancing copper extraction efficiency, particularly in light of the low recovery rates observed under similar conditions in this study. Additional studies have explored the use of alternative alcohols as hydrogen bond donors (HBDs). For instance, Liu et al. [16] employed ethylene glycol as the HBD in a DES and achieved copper recoveries of approximately 8% under identical conditions (25 °C, 25 min, with hydrogen peroxide). In contrast, Sabzkoohi et al. [7] reported a copper recovery of 75% using a DES formulated with ethylene glycol after 72 h of leaching at 85 °C, with iodine serving as the oxidizing agent. These findings suggest that the nature of the HBD, the choice of oxidant, and the operational parameters significantly influence the metal recovery performance of DES-based systems.

#### 3.2.2. Nickel Recovery in Binary DES

Nickel dissolution from electronic waste using deep eutectic solvents (DESs) has been investigated in recent studies. Oke et al. [24] reported nickel recoveries of 89% using a DES composed of choline chloride and dichloroacetic acid at 50 °C for 3 h, in the presence of hydrogen peroxide. These results demonstrate enhanced recovery efficiency under relatively mild conditions, attributed to the presence of chlorine in the acid and the oxidizing effect of hydrogen peroxide. Saffaj et al. [13] achieved nickel recoveries of 92% using a DES based on choline chloride and acetic acid, with iodine as the oxidizing agent, after 96 h at 65 °C. In comparison, the present study achieved similar recovery levels in a shorter timeframe, but at a higher temperature, and without the use of an oxidizing agent. Mishra et al. [22] reported nickel recoveries of 90% using a DES composed of choline chloride and formic acid, under conditions of 100 °C and 16 h. These results are comparable to those obtained in the present study, where 83% nickel recovery was achieved after 16 h at 90 °C.

To date, no studies have been reported on nickel recovery from PCBs using glycerin-based deep eutectic solvents (DESs). However, Sabzkoohi et al. [7] investigated the use of ethylene glycol, a type of alcohol, as the hydrogen bond donor (HBD) in a DES formulation. In that study, a nickel recovery of 45% was achieved after 24 h of leaching at 85 °C, with iodine serving as the oxidizing agent. This recovery rate is significantly higher than the 6% obtained in the present study under comparable conditions, highlighting the potential influence of both the HBD type and the presence of an oxidizing agent on nickel dissolution efficiency.

#### 3.2.3. Iron Recovery in Binary DES

The dissolution of iron from electronic waste using deep eutectic solvents (DESs) has been investigated in previous studies. Oke et al. [24] reported iron recoveries of 90% using a DES composed of choline chloride and trichloroacetic acid at 65 °C for 3 h, in the presence of hydrogen peroxide. This recovery rate is notably higher than that obtained in the present study, where 43% iron recovery was achieved after 2 h at a higher temperature. Similarly, Mishra et al. [22] reported iron recoveries of 90% using a DES formulated with choline chloride and formic acid, under conditions of 100 °C and 16 h of leaching. In comparison, the present study achieved a 62% iron recovery after 16 h at 90 °C using citric acid as the hydrogen bond donor, indicating a lower efficiency despite the relatively similar operational parameters.

#### 3.2.4. Zinc Recovery in Binary DES

The dissolution of zinc from electronic waste using deep eutectic solvents (DESs) has been investigated in previous studies. Mishra et al. [22] reported a zinc recovery of 90% using a urea-based DES after 21 h of leaching at 100 °C, which is comparable to the recoveries obtained in the present study using citric acid-based DES and the ternary system. Zhao et al. [30] achieved 90% zinc recovery employing a DES composed of choline chloride and glycolic acid, although specific operational conditions were not disclosed in the consulted source. Sabzkoohi et al. [7] reported a zinc recovery of 45% using ethaline as the DES, under conditions of 85 °C and 72 h, with iodine as the oxidizing agent. This contrasts with the present study, where a 40% zinc recovery was achieved after 24 h at 90 °C, without the use of any oxidizing agent. Popescu et al. [18] documented a zinc recovery of 95% from molten PCB alloys rich in tin, lead, and zinc, using a DES composed of choline chloride, ethylene glycol, and iodine as the oxidizing agent, under conditions of 75 °C and 168 h. These findings underscore the significant influence of DES composition, oxidant selection, and operational parameters on the efficiency of zinc recovery from electronic waste.

### 3.3. Leaching Test with a Ternary Deep Eutectic Solvent

Ternary deep eutectic solvents (DESs) offer operational advantages for metal leaching from complex matrices: lower viscosity and improved fluidity, lower melting points, and greater solubility and solvation capacity than binary formulations, which together reduce mass-transport limitations and enhance dissolution kinetics [25]. Based on these properties, the present study prioritized the use of the ternary DES composed of choline chloride, glycerol, and citric acid, whose efficacy has been reported for the recovery of critical metals from electronic waste. For instance, Aziz et al. [19] reported a silver recovery of up to 93% from PCBs using this ternary DES.

The leaching performance of the ternary deep eutectic solvent (DES) can be better understood by considering its key physical properties, particularly viscosity and density, which influence mass transfer, diffusion, and overall metal dissolution efficiency. According to published data, the viscosity of the choline chloride–citric acid (ChCl-CA) binary DES is relatively high, ranging between 1500 and 2500 mPa·s at 25 °C [8], whereas the choline chloride–glycerol (ChCl-GLY) system presents much lower values of 302–329 mPa·s at 25 °C [38]. Although no experimental viscosity data are available for the ternary choline chloride–glycerol–citric acid (ChCl-GLY-CA) system, a theoretical estimation based on the viscosities and relative proportions of the individual components suggests intermediate values of approximately 800–1000 mPa·s. These comparative values, together with the corresponding densities, are summarized in Table 3. Reported density values follow a similar trend, with 1.18 g mL^−1^ for ChCl-GLY, 1.21 g mL^−1^ for ChCl-GLY-CA, and 1.32 g mL^−1^ for ChCl-CA [19].

In this study, the ternary DES (ChCl-GLY-CA) achieved nearly identical recovery efficiencies to the ChCl-CA system (100% Cu and 99% Zn) while exhibiting a markedly lower viscosity. Based on these results and the comparative data summarized in Table 3, it can be concluded that the ternary DES provides more favorable operational conditions for metal leaching. The reduced viscosity significantly improves fluid dynamics and mass transfer, facilitating agitation and minimizing energy losses during mixing. In contrast, the citric acid–based DES (ChCl-CA) presented several operational challenges, including difficulties in filtration, agitation, and temperature control due to its high viscosity. Therefore, the ternary formulation represents a more practical and efficient medium for metal recovery under mild and scalable conditions.

Compared with the binary deep eutectic solvents (DESs) evaluated in this study—Glyceline (GLY, ChCl:glycerol) and Citrine (CA, ChCl:citric acid), the ternary DES (GLY-CA), composed of choline chloride, glycerol, and citric acid, exhibited superior performance for copper and zinc dissolution, achieving up to 99% recovery from multilayer ceramic capacitors (MLCCs) under the tested conditions. While Citrine (CA) also enabled high metal recovery, its significantly higher viscosity introduces operational challenges, particularly during filtration and agitation, which can hinder process efficiency. In contrast, GLY-CA combines the favorable extraction efficiency of CA with the low viscosity of GLY, resulting in a more manageable system. This lower viscosity enhances mass transfer, improves wetting, reduces energy consumption during agitation, and enables operation at moderate temperatures. Additionally, GLY-CA outperformed GLY alone, which yielded considerably lower recoveries for copper, zinc, nickel, and iron. The superior leaching performance and processability of GLY-CA are attributed to its synergistic physicochemical properties that optimize solvation, complexation, and mass transport of metal species, making it a promising formulation for efficient and scalable metal recovery.

### 3.4. Insights into the Function of Citric Acid in DES-Based Leaching

Although all three DESs exhibited leaching capability toward the metallic components of MLCCs, systems containing citric acid (ChCl-CA and ChCl-GLY-CA) achieved higher metal recovery efficiencies compared to ChCl-GLY. This behavior can be related to the chemical functionality of citric acid, whose role in promoting metal dissolution has been widely documented in aqueous leaching systems. Citric acid acts as both a proton donor and a complexing agent, enhancing metal solubilization through acid-driven dissolution and the formation of stable metal–citrate complexes [39]. Its three carboxylic acid groups and one hydroxyl group allow effective chelation of transition metal ions such as Cu^2+^, Ni^2+^, and Zn^2+^, while the acidification of the medium facilitates the breakdown of metal oxides and other compounds present in the MLCCs [40]. These mechanisms, well-established in aqueous systems, are also expected to operate within the DES environment.

When choline chloride is incorporated to form the ChCl-CA DES, additional synergistic effects arise that further enhance the leaching process. In this system, choline chloride acts as a hydrogen bond acceptor, while citric acid functions as a hydrogen bond donor, creating a stable hydrogen-bond network that defines the eutectic structure [8]. The addition of choline chloride promotes the formation of a liquid eutectic system with citric acid, providing a workable leaching medium without the need for water addition. [8]. Moreover, the chloride ions supplied by choline chloride can interact with transition metals to form soluble metal chloride complexes (e.g., CuCl_4_^2−^), which complement the metal–citrate complexation promoted by citric acid [22,40]. This dual mechanism, involving both chloride complexation and citrate chelation, facilitates more efficient metal dissolution and selectivity [40,41]. The eutectic environment further stabilizes the solvent and the resulting metal complexes, supporting enhanced recovery yields and demonstrating that the combination of choline chloride and citric acid provides chemical and operational advantages beyond those of citric acid alone [42].

The addition of glycerol to the ChCl-CA mixture to form the ternary DES (ChCl-CA-GLY) further modifies the physicochemical properties of the solvent, leading to improved leaching behavior. Glycerol acts as an additional hydrogen bond donor, strengthening the hydrogen-bond network within the eutectic system and thereby increasing its structural stability [8]. At the same time, the presence of glycerol significantly reduces the viscosity compared to the binary ChCl-CA DES, facilitating enhanced mass transfer and ionic mobility [43,44]. This reduction in viscosity improves the contact between the solid particles and the leaching medium, promoting faster metal dissolution under mild conditions. Furthermore, glycerol can participate in weak coordination interactions with metal ions, which, in combination with the chelating effect of citric acid and the chloride complexation from choline chloride, contribute to a balanced and efficient extraction mechanism [40,43,44]. These synergistic effects explain the comparable or even higher recoveries achieved with the ternary DES, demonstrating that the simultaneous presence of citric acid, choline chloride, and glycerol provides an optimal compromise between chemical reactivity and favorable physical properties. Consequently, the ternary DES represents a promising and more sustainable alternative for the selective recovery of metals from MLCCs.

### 3.5. Future Insights into Leaching with Ternary Deep Eutectic Solvents

While the present work demonstrates the potential of citric acid-based and ternary DESs for the selective recovery of metals from MLCCs, several aspects remain to be explored to achieve a deeper mechanistic understanding and optimize the process for practical applications. Further studies should focus on characterizing both the solid residues and the leachates to clarify dissolution pathways and complex formation mechanisms. Additionally, assessing the influence of oxidants and evaluating solvent reusability will be essential to establish the long-term efficiency and sustainability of DES-based leaching systems.

To further elucidate the leaching mechanisms and gain insights into the selectivity of the DES formulations, future studies should include the characterization of the solid residues remaining after leaching. Techniques such as X-ray photoelectron spectroscopy (XPS) or scanning electron microscopy coupled with energy-dispersive X-ray spectroscopy (SEM-EDS) could help identify which phases are preferentially dissolved and which persist. This would provide direct evidence supporting the observed selective metal recovery behavior.

Further studies could focus on the molecular characterization of the leachates using techniques such as FTIR, UV–Vis spectroscopy, or electrospray ionization mass spectrometry (ESI–MS) to identify and analyze metal–ligand complexes formed during leaching. This would enhance understanding of the coordination mechanisms responsible for selective metal recovery by deep eutectic solvents.

Although the current study focused on evaluating the leaching efficiency of different deep eutectic solvent systems in the absence of external oxidants, previous research has shown that the addition of oxidizing agents such as hydrogen peroxide (H_2_O_2_) or iodine (I_2_) can enhance metal recovery, particularly for transition metals like Fe and Ni [11,13,15,16,21,23]. Incorporating such agents could further improve extraction efficiencies but also introduce additional chemical complexity and environmental considerations. As such, while the use of oxidants was beyond the scope of this study, it presents a promising avenue for future research aimed at optimizing DES-based leaching processes for the recovery of electronic waste.

While this study focused on the initial leaching performance of citric acid-based DESs, future work should address the reversibility and reusability of these solvents. Investigating the potential for DES regeneration and metal recovery from leachates through techniques such as solvent extraction or electrochemical methods will be essential to evaluate the long-term viability and sustainability of the process. Assessing the performance of recycled DESs over multiple leaching cycles would also provide valuable insights into their stability and efficiency.

## 4. Materials and Methods

### 4.1. Dismantling of Post-Consumer Computers and Recovery of Multilayer Ceramic Capacitors

A total of twenty computers were dismantled, and their corresponding PCBs were extracted. The PCBs were subsequently subjected to a controlled thermal treatment using a Skil 8003 heat gun (SKIL Europe B.V., Breda, The Netherlands) to selectively recover multilayer ceramic capacitors (MLCCs), which constitute the primary focus of this study. MLCCs typically accounted for approximately 2% of the total mass of a PCB, which is consistent with findings reported in the literature for similar electronic devices (e.g., mobile phones, lighting equipment, or computers), as noted by Sideris et al. [3].

**Figure 5:**
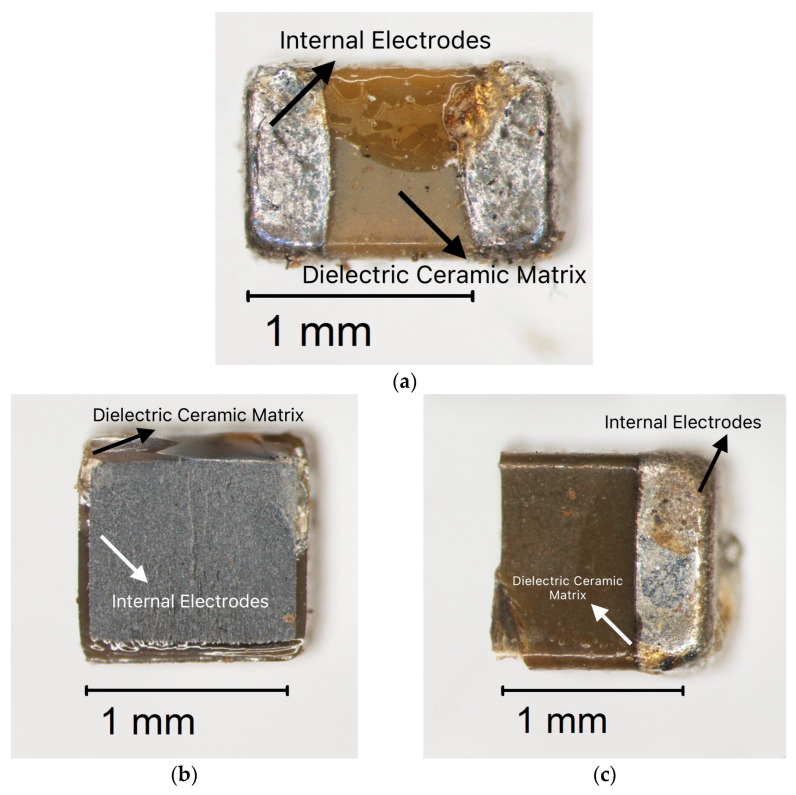
The multilayer ceramic capacitors (MLCCs) were dismantled from PCBs and examined under a trinocular stereoscope. (**a**) MLCC complete after dismantling, (**b**) Cross–sectional view of MLCCs, illustrating the dielectric ceramic matrix and the internal electrodes, (**c**) Surface view of MLCCs, showing the terminal electrodes and the composite ceramic matrix.

The dismantled multilayer ceramic capacitors (MLCCs) extracted from electronic boards are presented in Figure 5a–c. The images were captured using an Olympus SZX16 trinocular stereoscope equipped with an Olympus DP73 camera (Hachioji, Tokyo, Japan). This optical system enabled the acquisition of high-quality images, allowing for the identification of the external morphology of the devices (Figure 5a) and the arrangement of their metallic terminations (Figure 5b,c). This analysis represents a key preliminary step for further characterization and subsequent metal recovery processes.

### 4.2. Characterization of the Multilayer Ceramic Capacitors

The multilayer ceramic capacitors MLCCs recovered from computer PCBs were pulverized, homogenized, and quartered into 100 g fractions for subsequent experiments. X-ray fluorescence analysis was performed using the Bruker S8 Tiger instrument (Bruker, Karlsruhe, Germany) to determine the chemical composition of the material. X-ray diffraction was performed using the Bruker AXS D8 Advance model (Bruker, Karlsruhe, Germany) to determine the crystalline phase composition.

### 4.3. Preparation of Three Deep Eutectic Solvents Based on Choline Chloride

Three DESs were prepared: glyceline (GLY) (a binary mixture of choline chloride and glycerol), citrine (CA) (a binary mixture of choline chloride and citric acid), and a ternary (GLY-CA) mixture GLY-CA (choline chloride, glycerol, and citric acid). In these systems, choline chloride serves as the hydrogen bond acceptor (HBA), while both glycerol and citric acid serve as hydrogen bond donors (HBDs). The DESs were synthesized by mixing choline chloride (98% purity; Sigma Aldrich, Stinheim, Germany) with either citric acid (≥99% purity; Sigma Aldrich, Stinheim, Germany), glycerol (99.5% purity; Sigma Aldrich, Stinheim, Germany), or a combination of both, at the molar ratios specified in Table 4, based on the proportions reported by Aziz et al. [19]. All mixtures were maintained under constant stirring at 80 °C until a clear homogeneous liquid was formed, followed by cooling to room temperature (15 °C) before use.

### 4.4. Leaching Tests of Multilayer Ceramic Capacitors Using Deep Eutectic Solvents

Each leaching test was performed using 0.2 g of pulverized multilayer ceramic capacitors (MLCCs) in 10 g of DES (glyceline (GLY), citrine (CA), or ternary (GLY-CA)), under controlled conditions of temperature (90 °C) and agitation (400 rpm). The temperature was maintained at 90 °C using a thermostatically controlled glycerol bath. During the experiments, 200 μL aliquots were collected at 0.5, 1, 2, 4, 6, and 24 h. The samples were then filtered and diluted in 50 mL volumetric flasks with a 2% HCl solution and subsequently analyzed by atomic absorption spectroscopy (AAS) using a PerkinElmer AA 300 instrument (PerkinElmer, Shelton, CT, USA) to quantify the dissolved metals, namely copper (Cu), nickel (Ni), iron (Fe), and zinc (Zn).

## 5. Conclusions

Multilayer ceramic capacitors (MLCCs) were extracted from post-consumer computers, and their characterization confirmed the coexistence of ceramic and metallic phases. The ceramic matrix was primarily composed of barium titanate (BaTiO_3_) and barium iron oxide (BaFeO_3−x_), with magnetite (Fe_3_O_4_) identified as a secondary phase. The metallic phases consisted of Cu–Ni and Cu–Zn alloys, found in the internal electrodes and external terminations. Elemental analysis revealed barium (45.8%), titanium (14.8%), and nickel (16.1%) as the major constituents, while iron (5.8%), copper (2.5%), zinc (1.5%), and tin (3.8%) were present in smaller amounts.

Leaching experiments using three choline chloride-based deep eutectic solvents (DESs), Glyceline-GLY (choline chloride and glycerol), Citrine-CA (choline chloride and citric acid), and the ternary system GLY-CA (choline chloride, glycerol, and citric acid), demonstrated distinct differences in metal recovery efficiency from multilayer ceramic capacitors (MLCCs). Copper and zinc were optimally recovered using both Citrine and the ternary DES, with each achieving a recovery rate of up to 99% after 24 h at 90 °C. Zinc, in particular, exhibited rapid dissolution kinetics in the ternary system, achieving ~90% recovery within the first hour and stabilizing at nearly 100%. For nickel, Citrine outperformed the other systems, achieving 99% recovery, followed by GLY-CA (25%) and GLY (6%). Iron exhibited the lowest recovery across all systems, with Citrine reaching 68%, GLY-CA 44%, and GLY only 16%. This indicates that under the tested conditions, iron is less efficiently leached using these DESs.

The ternary DES (GLY-CA) demonstrated clear operational and chemical advantages over the binary systems. By combining citric acid (from Citrine) with glycerol (from Glyceline), the ternary system retained high metal recovery efficiencies for Cu, Zn, and, to a lesser extent, Ni, while significantly reducing the high viscosity associated with Citrine at lower temperatures. Therefore, the incorporation of GLY-CA provides a more practical and scalable alternative for metal recovery processes.

## Figures and Tables

**Table 1:** Chemical characterization of the multilayer ceramic capacitors.

Element	Content (%)
Ba	45.8
Ni	16.1
Ti	14.8
Fe	5.8
Sn	3.8
Cu	2.5
Zn	1.5
Others	9.7

**Table 2:** Crystalline Phase Characterization of the Multilayer Ceramic Capacitors.

Mineral	Formula	Abundance
Barium Titanium Oxide	BaTiO_3_	+++
Barium Iron Oxide	BaFeO_3−x_	+++
Magnetite	Fe_3_O_4_	++
Copper Zinc	CuZn_5_	+
Copper Nickel	Cu_0.81_Ni_0.19_	+

(+) Low abundance; (++) Moderate abundance; (+++) High abundance.

**Table 3:** Comparative physical properties (viscosity and density) of the deep eutectic solvents (DESs) used in this study.

DES System	Molar Ratio	Viscosity (mPa·s) 25 °C	Density (g mL^−1^) 25 °C
ChCl-GLY	1:2	302–319 [38]	1.18 [19]
ChCl-CA	1:1	1500–2500 [8]	1.32 [19]
ChCl-GLY-CA	2.4:3.4:1	800–1000 *	1.21 [19]

* Viscosity value for the ternary ChCl-GLY-CA DES was theoretically estimated based on literature data for individual binary systems and relative component proportions.

**Table 4:** Composition and molar ratios of the prepared deep eutectic solvents (DESs).

DES Code	Components (HBA:HBD)	Molar Ratio (ChCl:HBD)
GLY (Glyceline)	Choline chloride/glycerol	1:2
CA (Citrine)	Choline chloride/citric acid	1:1
GLY-CA (Ternary)	Choline chloride/glycerol/citric acid	2.4:3.4:1

## Data Availability

The original contributions presented in this study are included in the article. Further inquiries can be directed to the corresponding author.

## Abbreviations

The following abbreviations are used in this manuscript:

MLCCs	Multilayer Ceramic Capacitors
PCBs	Printed circuit boards
DES	Deep eutectic solvent
ChCl	Choline Chloride
CA	Citric acid
GLY	Glycerol
GLY-CA	Ternary DES composed of choline chloride, citric acid, and glycerol.
WEEE	Waste electrical and electronic equipment
HBDs	Hydrogen bond donors
HBAs	Hydrogen bond acceptors
TDES	Ternary deep eutectic solvents
BDES	Binary deep eutectic solvents
XRF	X-ray fluorescence
